# Alternariol 9-*O*-methyl ether

**DOI:** 10.1107/S1600536812015000

**Published:** 2012-04-21

**Authors:** Sreekanth Dasari, Mohan Bhadbhade, Brett A. Neilan

**Affiliations:** a School of Biotechnology and Biomolecular Sciences, University of New South Wales, Sydney, 2052 NSW, Australia; b Mark Wainwright Analytical Centre, University of New South Wales, Sydney, 2052 NSW, Australia

## Abstract

The title compound (AME; systematic name: 3,7-dihy­droxy-9-meth­oxy-1-methyl-6*H*-benzo[*c*]chromen-6-one), C_15_H_12_O_5_, was isolated from an endophytic fungi *Alternaria* sp., from *Catharanthus roseus* (common name: Madagascar periwinkle). There is an intramolecular O—H⋯O hydrogen bond in the essentially planar mol­ecule (r.m.s. deviation 0.02 Å). In the crystal, the molecule forms an O—H⋯O hydrogen bond with its centrosymmetric counterpart with four bridging inter­actions (two O—H⋯O and two C—H⋯O). The almost planar sheets of the dimeric units thus formed are stacked along *b* axis *via* C—H⋯π and π–π contacts [with C⋯C short contacts between aromatic moieties of 3.324 (3), 3.296 (3) and 3.374 (3) Å].

## Related literature

Species of the fungal genus *Alternaria* are known producers of mycotoxins and have previously been described as plant endophytes. For the isolation of Alternariol (AOH) and Alternariol 9-*O*-methyl ether (AME) see: An *et al.* (1989 ▶); Wen (2009 ▶); Ashour *et al.* (2011 ▶). For ^1^H, ^13^C and two-dimensional experimental data analysis see: Koch *et al.* (2005 ▶); Siegel *et al.* (2010 ▶). For the biological activity see: Aly *et al.* (2008 ▶).
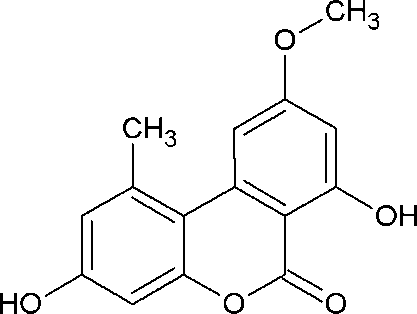


## Experimental

### 

#### Crystal data

C_15_H_12_O_5_
*M* *_r_* = 272.25
Triclinic, 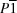

*a* = 7.1819 (7) Å
*b* = 8.9393 (8) Å
*c* = 10.2511 (10) Å
α = 105.296 (5)°
β = 105.174 (4)°
γ = 101.430 (4)°
*V* = 586.90 (10) Å^3^
*Z* = 2
Mo *K*α radiation
μ = 0.12 mm^−1^
*T* = 160 K
0.29 × 0.13 × 0.06 mm

#### Data collection

Bruker Kappa APEXII CCD diffractometer
Absorption correction: multi-scan (*SADABS*; Bruker, 2001 ▶) *T*_min_ = 0.967, *T*_max_ = 0.993
7824 measured reflections
2062 independent reflections
1718 reflections with *I* > 2σ(*I*)
*R*_int_ = 0.020

#### Refinement

*R*[*F* ^2^ > 2σ(*F* ^2^)] = 0.033
*wR*(*F* ^2^) = 0.096
*S* = 1.05
2062 reflections
184 parameters
H-atom parameters constrained
Δρ_max_ = 0.25 e Å^−3^
Δρ_min_ = −0.20 e Å^−3^

### 

Data collection: *APEX2* (Bruker, 2007 ▶); cell refinement: *SAINT* (Bruker, 2007 ▶); data reduction: *SAINT*; program(s) used to solve structure: *SHELXS97* (Sheldrick, 2008 ▶); program(s) used to refine structure: *SHELXL97* (Sheldrick, 2008 ▶); molecular graphics: *SHELXTL-Plus* (Sheldrick, 2008 ▶); software used to prepare material for publication: *SHELXL97*.

## Supplementary Material

Crystal structure: contains datablock(s) I, global. DOI: 10.1107/S1600536812015000/hg5207sup1.cif

Structure factors: contains datablock(s) I. DOI: 10.1107/S1600536812015000/hg5207Isup2.hkl

Additional supplementary materials:  crystallographic information; 3D view; checkCIF report

## Figures and Tables

**Table 1:** Hydrogen-bond geometry (Å, °)

*D*—H⋯*A*	*D*—H	H⋯*A*	*D*⋯*A*	*D*—H⋯*A*
O4—H4⋯O4^i^	0.82	2.47	2.9371 (19)	117
C9—H9⋯O1^ii^	0.93	2.64	3.4645 (16)	148
C4—H4*A*⋯O2^iii^	0.93	2.32	3.2511 (16)	174
O4—H4⋯O3	0.82	1.84	2.5692 (13)	148
O1—H1⋯O3^iii^	0.82	2.14	2.9619 (13)	176

Symmetry codes: (i) 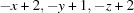; (ii) 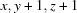; (iii) 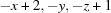.

**Fig. 1.**
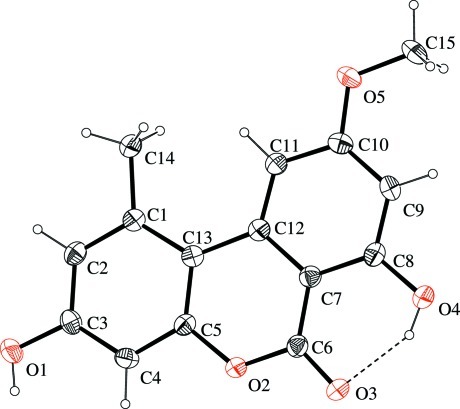
ORTEP view of the title compound showing an intra molecular O—H···O hydrogen bond.. (Thermal ellipsoids are drawn at 40% probability level).

**Fig. 2.**
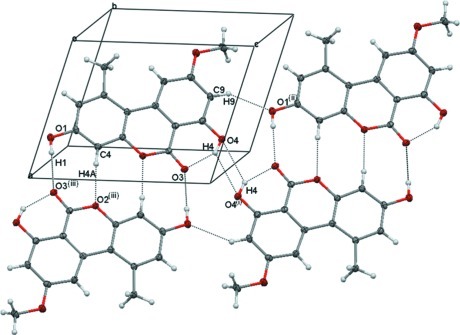
Molecular association forming planar sheets in ac plane via network of O—H···O and C—H···O contacts. Symmetry codes: (i) -x + 2, -y + 1, -z + 2; (ii) x, y + 1, z + 1; (iii) -x + 2, -y, -z + 1.

**Fig. 3.**
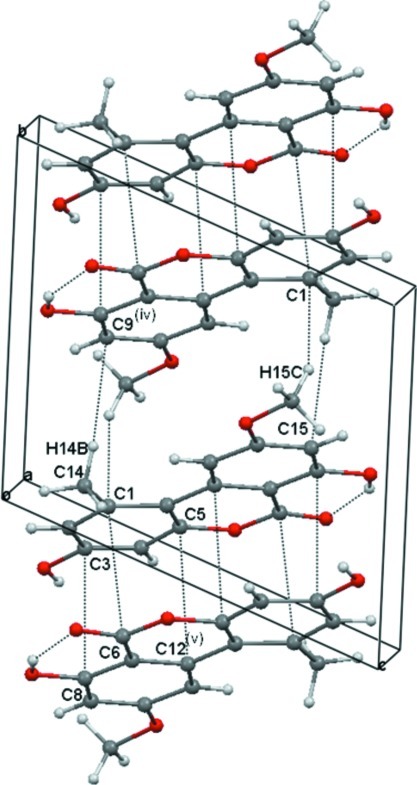
: Packing of molecules along b axis making C—H···π and π–π interactions. Symmetry codes: (iv) 1 - x, 1 - y, 1 - z; (v) 1 - x, -y, 1 - z.

## supplementary crystallographic information

### Comment

The title compound was isolated from an endophytic *Alternaria* sp. from the host plant *Catharanthus roseus*. Alternariol 9-*O*-methyl ether is a mycotoxin often isolated from *Alternaria* sp.. It has been reported to exhibit mild antimicrobial activity (An *et al.*, 1989; Wen 2009; Ashour *et al.*, 2011), cytotoxicity and protein kinase inhibitory activity (Aly *et al.*, 2008). ^1^H, ^13^C and two-dimensional NMR spectral data was reported previously (Koch *et al.*, 2005; Siegel *et al*., 2010). Although mycotoxins have been studied extensively, the single-crystal structure of alternariol 9-*O*-methy ether is reported here for the first time.

An *ORTEP* view of the title compound (Fig. 1) shows an intramolecular O4—H4···O3 hydrogen bond. The two centrosymmetric partners make a total of four interactions - the two hydrogen bonds O1—H1···O3^(iii)^ and C4—H4A···O2^(iii)^ , ((iii) -*x* + 2, -*y*, -*z* + 1) are duplicated across the inversion centre. The hydrogen H4 also makes a short contact with O4 of another centrosymmetrically related molecule (O4—H4···O4^(i)^, (i) -*x* + 2, -*y* + 1, -*z* + 2). The bridged centrosymmetric dimers translated along *c* axis make C9—H9···O1^(ii)^, (ii) *x*, *y* + 1, *z* + 1 and O4—H4···O4^(i)^ contacts (Table. 1 and Fig. 2). These almost planar sheets thus formed in *ac* plane are stacked along *b* axis; these make C—H···π contacts with the edge of the ring in one direction (C14—H14B···C9^(iv)^ = 2.88 Å and C15—H15C···C1^(iv)^ = 2.85 Å, (iv) 1 - *x*, 1 - *y*, 1 - *z*) and π–π contacts in the opposite direction with considerable overlap of aromatic moieties with values of short contact between C3···C8^(v)^ = 3.324 (3) Å, C1···C6^(v)^ = 3.296 (3) Å and C5···C12^(v)^ = 3.374 (3) Å with (v) 1 - *x*, -*y*, 1 - *z* (Fig. 3).

### Experimental

*Alternaria* sp. was isolated from *Catharanthus roseus* and cultured in malt extract media for production of secondary metabolites. The crude ethyl acetate extract was fractionated on silica gel with a stepwise gradient of hexane to ethyl acetate and to methanol to yield 12 fractions. Fractions 6 and 7, which were eluted with 2:1 and 1:1 hexane, ethyl acetate showed fine needle like crystals on slow evaporation. These fine needles were recrystallized using dichloromethane to yield plate like crystals for crystallographic analysis. The positive and negative ESI-MS analysis of the title compound exhibited molecular ion peak at *m/z* 273, attributing to [*M*+H]^+^ and at *m/z* 271, attributing to [M—H]^-^ , respectively, infereing its molecular weight to be 272 g/mol which is in agreement with the previously reported values (Ashour *et al.*, 2011).

### Refinement

H atoms were positioned geometrically with C—H = 0.93 — 0.96 Å and O—H = 0.82 Å. *U*_iso_(H) values were set at 1.2*U*eq (aromatic) or 1.5*U*eq of the parent atom (methyl group).

### Crystal data

**Table:**

C_15_H_12_O_5_	*Z* = 2
*M**_r_* = 272.25	*F*(000) = 284
Triclinic, *P*1	*D*_x_ = 1.541 Mg m^−^^3^
Hall symbol: -P 1	Mo *K*α radiation, λ = 0.71073 Å
*a* = 7.1819 (7) Å	Cell parameters from 4112 reflections
*b* = 8.9393 (8) Å	θ = 2.7–29.6°
*c* = 10.2511 (10) Å	µ = 0.12 mm^−^^1^
α = 105.296 (5)°	*T* = 160 K
β = 105.174 (4)°	Plates, colourless
γ = 101.430 (4)°	0.29 × 0.13 × 0.06 mm
*V* = 586.90 (10) Å^3^	

### Data collection

**Table:**

Bruker Kappa APEXII CCD diffractometer	2062 independent reflections
Radiation source: fine-focus sealed tube	1718 reflections with *I* > 2σ(*I*)
Graphite monochromator	*R*_int_ = 0.020
φ scans, and ω scans with κ offsets	θ_max_ = 25.0°, θ_min_ = 2.5°
Absorption correction: multi-scan (*SADABS*; Bruker, 2001)	*h* = −8→8
*T*_min_ = 0.967, *T*_max_ = 0.993	*k* = −10→10
7824 measured reflections	*l* = −12→12

### Refinement

**Table:**

Refinement on *F*^2^	Primary atom site location: structure-invariant direct methods
Least-squares matrix: full	Secondary atom site location: difference Fourier map
*R*[*F*^2^ > 2σ(*F*^2^)] = 0.033	Hydrogen site location: inferred from neighbouring sites
*wR*(*F*^2^) = 0.096	H-atom parameters constrained
*S* = 1.05	*w* = 1/[σ^2^(*F*_o_^2^) + (0.0511*P*)^2^ + 0.1544*P*] where *P* = (*F*_o_^2^ + 2*F*_c_^2^)/3
2062 reflections	(Δ/σ)_max_ < 0.001
184 parameters	Δρ_max_ = 0.25 e Å^−^^3^
0 restraints	Δρ_min_ = −0.20 e Å^−^^3^

### Special details

**Table:**

Geometry. All e.s.d.'s (except the e.s.d. in the dihedral angle between two l.s. planes) are estimated using the full covariance matrix. The cell e.s.d.'s are taken into account individually in the estimation of e.s.d.'s in distances, angles and torsion angles; correlations between e.s.d.'s in cell parameters are only used when they are defined by crystal symmetry. An approximate (isotropic) treatment of cell e.s.d.'s is used for estimating e.s.d.'s involving l.s. planes.
Refinement. Refinement of *F*^2^ against ALL reflections. The weighted *R*-factor *wR* and goodness of fit *S* are based on *F*^2^, conventional *R*-factors *R* are based on *F*, with *F* set to zero for negative *F*^2^. The threshold expression of *F*^2^ > σ(*F*^2^) is used only for calculating *R*-factors(gt) *etc*. and is not relevant to the choice of reflections for refinement. *R*-factors based on *F*^2^ are statistically about twice as large as those based on *F*, and *R*- factors based on ALL data will be even larger.

### Fractional atomic coordinates and isotropic or equivalent isotropic displacement parameters (Å^2^)

**Table:**

	*x*	*y*	*z*	*U*_iso_*/*U*_eq_	
O1	0.65936 (15)	−0.18780 (12)	0.08182 (10)	0.0292 (3)	
H1	0.7594	−0.2138	0.1163	0.044*	
O2	0.84646 (14)	0.13308 (12)	0.56397 (9)	0.0245 (3)	
O3	0.96545 (14)	0.26527 (12)	0.79381 (10)	0.0267 (3)	
O4	0.78595 (15)	0.45323 (13)	0.91399 (10)	0.0329 (3)	
H4	0.8735	0.4077	0.9083	0.049*	
O5	0.17885 (16)	0.48775 (13)	0.61731 (11)	0.0331 (3)	
C1	0.4041 (2)	0.07774 (16)	0.25561 (14)	0.0202 (3)	
C2	0.4514 (2)	−0.03110 (16)	0.15399 (14)	0.0225 (3)	
H2	0.3642	−0.0722	0.0598	0.027*	
C3	0.6240 (2)	−0.08085 (16)	0.18775 (14)	0.0219 (3)	
C4	0.7533 (2)	−0.02073 (16)	0.32735 (14)	0.0223 (3)	
H4A	0.8701	−0.0517	0.3529	0.027*	
C5	0.7053 (2)	0.08647 (16)	0.42815 (14)	0.0201 (3)	
C6	0.8305 (2)	0.23648 (16)	0.67963 (14)	0.0208 (3)	
C7	0.66319 (19)	0.30221 (15)	0.66141 (14)	0.0200 (3)	
C8	0.6471 (2)	0.41025 (16)	0.78306 (14)	0.0232 (3)	
C9	0.4878 (2)	0.47638 (16)	0.77364 (14)	0.0244 (3)	
H9	0.4787	0.5483	0.8542	0.029*	
C10	0.3421 (2)	0.43228 (16)	0.64067 (15)	0.0241 (3)	
C11	0.3534 (2)	0.32476 (17)	0.51851 (14)	0.0249 (3)	
H11	0.2522	0.2980	0.4311	0.030*	
C12	0.5112 (2)	0.25710 (15)	0.52416 (14)	0.0197 (3)	
C13	0.5353 (2)	0.14195 (16)	0.40140 (14)	0.0197 (3)	
C14	0.2119 (2)	0.11934 (18)	0.20211 (14)	0.0267 (3)	
H14A	0.1482	0.0608	0.1014	0.040*	
H14B	0.2416	0.2335	0.2180	0.040*	
H14C	0.1232	0.0903	0.2528	0.040*	
C15	0.1611 (2)	0.60559 (18)	0.73528 (16)	0.0309 (4)	
H15A	0.1602	0.5618	0.8112	0.046*	
H15B	0.0380	0.6330	0.7045	0.046*	
H15C	0.2733	0.7010	0.7693	0.046*	

### Atomic displacement parameters (Å^2^)

**Table:**

	*U*^11^	*U*^22^	*U*^33^	*U*^12^	*U*^13^	*U*^23^
O1	0.0335 (6)	0.0342 (6)	0.0220 (5)	0.0176 (5)	0.0108 (4)	0.0050 (4)
O2	0.0216 (5)	0.0295 (5)	0.0189 (5)	0.0119 (4)	0.0028 (4)	0.0029 (4)
O3	0.0210 (5)	0.0307 (6)	0.0217 (5)	0.0091 (4)	0.0008 (4)	0.0033 (4)
O4	0.0267 (6)	0.0418 (6)	0.0203 (5)	0.0153 (5)	0.0002 (4)	−0.0020 (4)
O5	0.0346 (6)	0.0396 (6)	0.0266 (5)	0.0252 (5)	0.0080 (4)	0.0050 (5)
C1	0.0205 (7)	0.0210 (7)	0.0199 (7)	0.0052 (5)	0.0071 (5)	0.0084 (5)
C2	0.0224 (7)	0.0254 (7)	0.0178 (7)	0.0049 (6)	0.0046 (5)	0.0075 (6)
C3	0.0261 (7)	0.0211 (7)	0.0211 (7)	0.0070 (6)	0.0121 (6)	0.0071 (6)
C4	0.0204 (7)	0.0251 (7)	0.0245 (7)	0.0100 (6)	0.0086 (6)	0.0095 (6)
C5	0.0188 (7)	0.0223 (7)	0.0180 (7)	0.0049 (5)	0.0045 (5)	0.0069 (5)
C6	0.0188 (7)	0.0203 (7)	0.0204 (7)	0.0032 (5)	0.0055 (6)	0.0046 (6)
C7	0.0185 (7)	0.0194 (7)	0.0212 (7)	0.0041 (5)	0.0060 (6)	0.0067 (6)
C8	0.0216 (7)	0.0227 (7)	0.0203 (7)	0.0033 (6)	0.0039 (6)	0.0044 (6)
C9	0.0278 (8)	0.0215 (7)	0.0228 (7)	0.0090 (6)	0.0098 (6)	0.0031 (6)
C10	0.0239 (7)	0.0238 (7)	0.0278 (7)	0.0112 (6)	0.0090 (6)	0.0102 (6)
C11	0.0259 (7)	0.0282 (8)	0.0188 (7)	0.0123 (6)	0.0037 (6)	0.0055 (6)
C12	0.0198 (7)	0.0187 (7)	0.0205 (7)	0.0045 (5)	0.0064 (5)	0.0075 (6)
C13	0.0197 (7)	0.0200 (7)	0.0202 (7)	0.0054 (5)	0.0069 (5)	0.0080 (6)
C14	0.0243 (8)	0.0326 (8)	0.0191 (7)	0.0106 (6)	0.0037 (6)	0.0040 (6)
C15	0.0335 (8)	0.0301 (8)	0.0333 (8)	0.0185 (7)	0.0152 (7)	0.0065 (7)

### Geometric parameters (Å, º)

**Table:**

O1—C3	1.3602 (16)	C5—C13	1.3958 (19)
O1—H1	0.8200	C6—C7	1.4299 (19)
O2—C6	1.3446 (16)	C7—C8	1.4090 (18)
O2—C5	1.3884 (15)	C7—C12	1.4338 (18)
O3—C6	1.2344 (16)	C8—C9	1.382 (2)
O4—C8	1.3486 (16)	C9—C10	1.3835 (19)
O4—H4	0.8200	C9—H9	0.9300
O5—C10	1.3508 (17)	C10—C11	1.3955 (19)
O5—C15	1.4317 (16)	C11—C12	1.3819 (19)
C1—C2	1.3889 (19)	C11—H11	0.9300
C1—C13	1.4305 (18)	C12—C13	1.4743 (18)
C1—C14	1.5031 (19)	C14—H14A	0.9600
C2—C3	1.3890 (19)	C14—H14B	0.9600
C2—H2	0.9300	C14—H14C	0.9600
C3—C4	1.3784 (18)	C15—H15A	0.9600
C4—C5	1.3785 (19)	C15—H15B	0.9600
C4—H4A	0.9300	C15—H15C	0.9600
			
C3—O1—H1	109.5	C8—C9—C10	117.96 (12)
C6—O2—C5	122.41 (10)	C8—C9—H9	121.0
C8—O4—H4	109.5	C10—C9—H9	121.0
C10—O5—C15	118.10 (11)	O5—C10—C9	123.74 (12)
C2—C1—C13	119.78 (12)	O5—C10—C11	114.42 (12)
C2—C1—C14	116.06 (11)	C9—C10—C11	121.84 (12)
C13—C1—C14	124.16 (12)	C12—C11—C10	121.63 (12)
C1—C2—C3	122.49 (12)	C12—C11—H11	119.2
C1—C2—H2	118.8	C10—C11—H11	119.2
C3—C2—H2	118.8	C11—C12—C7	116.99 (12)
O1—C3—C4	122.22 (12)	C11—C12—C13	125.49 (12)
O1—C3—C2	118.81 (12)	C7—C12—C13	117.51 (12)
C4—C3—C2	118.97 (12)	C5—C13—C1	114.81 (12)
C3—C4—C5	118.40 (12)	C5—C13—C12	117.24 (12)
C3—C4—H4A	120.8	C1—C13—C12	127.96 (12)
C5—C4—H4A	120.8	C1—C14—H14A	109.5
C4—C5—O2	111.77 (11)	C1—C14—H14B	109.5
C4—C5—C13	125.54 (12)	H14A—C14—H14B	109.5
O2—C5—C13	122.68 (12)	C1—C14—H14C	109.5
O3—C6—O2	115.38 (12)	H14A—C14—H14C	109.5
O3—C6—C7	125.97 (12)	H14B—C14—H14C	109.5
O2—C6—C7	118.65 (11)	O5—C15—H15A	109.5
C8—C7—C6	118.38 (11)	O5—C15—H15B	109.5
C8—C7—C12	120.11 (12)	H15A—C15—H15B	109.5
C6—C7—C12	121.49 (12)	O5—C15—H15C	109.5
O4—C8—C9	116.98 (12)	H15A—C15—H15C	109.5
O4—C8—C7	121.56 (12)	H15B—C15—H15C	109.5
C9—C8—C7	121.46 (12)		
			
C13—C1—C2—C3	−0.4 (2)	C15—O5—C10—C11	−176.59 (12)
C14—C1—C2—C3	−179.96 (13)	C8—C9—C10—O5	179.73 (13)
C1—C2—C3—O1	−179.96 (12)	C8—C9—C10—C11	−0.1 (2)
C1—C2—C3—C4	0.3 (2)	O5—C10—C11—C12	179.96 (12)
O1—C3—C4—C5	−179.60 (12)	C9—C10—C11—C12	−0.2 (2)
C2—C3—C4—C5	0.2 (2)	C10—C11—C12—C7	0.1 (2)
C3—C4—C5—O2	178.48 (11)	C10—C11—C12—C13	179.58 (12)
C3—C4—C5—C13	−0.5 (2)	C8—C7—C12—C11	0.4 (2)
C6—O2—C5—C4	−179.66 (12)	C6—C7—C12—C11	178.63 (12)
C6—O2—C5—C13	−0.6 (2)	C8—C7—C12—C13	−179.21 (12)
C5—O2—C6—O3	179.01 (11)	C6—C7—C12—C13	−0.93 (19)
C5—O2—C6—C7	−0.80 (19)	C4—C5—C13—C1	0.4 (2)
O3—C6—C7—C8	0.1 (2)	O2—C5—C13—C1	−178.50 (11)
O2—C6—C7—C8	179.88 (12)	C4—C5—C13—C12	−179.87 (12)
O3—C6—C7—C12	−178.21 (13)	O2—C5—C13—C12	1.2 (2)
O2—C6—C7—C12	1.6 (2)	C2—C1—C13—C5	0.05 (19)
C6—C7—C8—O4	0.7 (2)	C14—C1—C13—C5	179.61 (12)
C12—C7—C8—O4	179.02 (12)	C2—C1—C13—C12	−179.65 (12)
C6—C7—C8—C9	−179.00 (13)	C14—C1—C13—C12	−0.1 (2)
C12—C7—C8—C9	−0.7 (2)	C11—C12—C13—C5	−179.96 (13)
O4—C8—C9—C10	−179.16 (12)	C7—C12—C13—C5	−0.44 (19)
C7—C8—C9—C10	0.5 (2)	C11—C12—C13—C1	−0.3 (2)
C15—O5—C10—C9	3.5 (2)	C7—C12—C13—C1	179.25 (12)

### Hydrogen-bond geometry (Å, º)

**Table:**

*D*—H···*A*	*D*—H	H···*A*	*D*···*A*	*D*—H···*A*
O4—H4···O4^i^	0.82	2.47	2.9371 (19)	117
C9—H9···O1^ii^	0.93	2.64	3.4645 (16)	148
C4—H4*A*···O2^iii^	0.93	2.32	3.2511 (16)	174
O4—H4···O3	0.82	1.84	2.5692 (13)	148
O1—H1···O3^iii^	0.82	2.14	2.9619 (13)	176

Symmetry codes: (i) −*x*+2, −*y*+1, −*z*+2; (ii) *x*, *y*+1, *z*+1; (iii) −*x*+2, −*y*, −*z*+1.

## References

[bb1] Aly, H. A., Edrasa-Ebel, R., Indrani, D. I., Wray, V., Muller, G. E. W., Totzke, F., Zirrgiebel, U., Schachtele, C., Kubbutat, G. H. M., Lin, W. H., Proksch, P. & Ebel, R. (2008). *J. Nat. Prod.* **71**, 972–980. 10.1021/np070447m 18494522

[bb2] An, Y., Zhao, T., Miao, J., Liu, G., Zheng, Y., Xu, Y. & Van Etten, L. R. (1989). *J. Agric. Food Chem.* **37**, 1341–1343.

[bb3] Ashour, M., Yehia, M. H. & Proksch, P. (2011). *J. Nat. Prod. India*, **4**, 108–114.

[bb4] Bruker (2001). *SADABS* Bruker AXS Inc., Madison, Wisconsin, USA.

[bb5] Bruker (2007). *APEX2* and *SAINT* Bruker AXS Inc., Madison, Wisconsin, USA.

[bb6] Koch, K., Podlech, J., Pfeiffer, E. & Metzler, M. (2005). *J. Org. Chem.* **70**, 3275–3276. 10.1021/jo050075r 15822993

[bb7] Sheldrick, G. M. (2008). *Acta Cryst.* A**64**, 112–122. 10.1107/S0108767307043930 18156677

[bb8] Siegel, D., Troyanov, S., Noack, J., Emmerling, F. & Nehls, I. (2010). *Acta Cryst.* E**66**, o1366. 10.1107/S1600536810017502 PMC2979420 21579450

[bb9] Wen, G. (2009). *World J. Microbiol. Biotechnol.* **25**, 1677–1683.

